# Retraction: A Priori Activation of Apoptosis Pathways of Tumor (AAAPT) technology: Development of targeted apoptosis initiators for cancer treatment

**DOI:** 10.1371/journal.pone.0287148

**Published:** 2023-06-08

**Authors:** The PLOS ONE Editors

Following the publication of this article [1], concerns were raised regarding results presented in Figs 5, 6, 8, S4 Fig, and S1 File. Specifically,

- The Fig 5C Bcl-2 and β-actin panels appear similar when color levels are adjusted to visualize the background.
- The Fig 6B 5µM (right) panel appears similar to the Fig 6B 10µM (left) panel.
- The Fig 8A AMP-001 GAPDH and AMP-002 GAPDH panels appear similar.
- There are concerns with the underlying blot data presented in the published S1 File:
  ○ There appears to be a region in lanes 1 and 2 in the β-actin results presented in the underlying data for the Fig 5C panels which does not appear to match the remainder of the blot, suggestive of inappropriate image manipulation.
  ○ The underlying data provided in S1 File for Fig 5C do not appear to match the corresponding panels presented in Fig 5C.
  ○ In the underlying data provided for Fig 5C, the sizes for the Bax and the β-actin results are both marked as 20kDa, despite their different locations on the blot.
  ○ There appears to be a region in lanes 1–4 in the total PARP results presented in the underlying data for the Fig 8B panels which does not appear to match lane 5, suggestive of inappropriate image manipulation.
  ○ The underlying data provided in S1 File for Figs 8A and 8B does not appear to match the corresponding panels presented in Fig 8.
- The following S4 Fig panels appear to partially overlap:
  ○ The first TMD231 + 5 uM AMP-001 image (page 2, top right) and the third TMD231 + 5 uM AMP-001 image (page 2, middle right).
  ○ The first TMD231 + 10 uM AMP-001 image (page 2, bottom right) and the third TMD231 + 10 uM AMP-001 image (page 3, top right).

The corresponding author did not provide a clarification for the above concerns involving Figs 5, 8, and S1 File.

The last author clarified that the panel duplication in Fig 6 was the result of an inadvertent image duplication introduced during the preparation of this figure, and the authors provided a replacement figure. The last author also clarified that the partially overlapping panels in S4 Fig represent triplicate data obtained from the same plates of cells, and apologized for the inadvertent errors in the published Fig 6.

In light of the concerns affecting multiple figure panels and underlying data that question the integrity of the data presented in Figs 5 and 8, and the S1 File, the *PLOS ONE* Editors retract this article.

CM agreed with the retraction. RSP and MT did not agree with the retraction. HN responded but neither agreed nor disagreed with the retraction decision. STV, RP, SR, and MA either did not respond directly or could not be reached.
